# The Triple Burden of Tuberculosis, Human Immunodeficiency Virus and Silicosis among Artisanal and Small-Scale Miners in Zimbabwe

**DOI:** 10.3390/ijerph192113822

**Published:** 2022-10-24

**Authors:** Dingani Moyo, Ronald Ncube, Fungai Kavenga, Lilian Chikwava, Tawanda Mapuranga, Nathan Chiboyiwa, Chipo Chimunhu, Frank Mudzingwa, Orippa Muzvidziwa, Petronella Ncube, Tariro Christwish Mando, Florence Moyo, Blessings Chigaraza, Hellen Masvingo, Collins Timire

**Affiliations:** 1 Baines Occupational Health Services, Harare 024, Zimbabwe; 2 Occupational Health Division, School of Public Health, University of the Witwatersrand, Johannesburg 2000, South Africa; 3 Faculty of Medicine and Health Sciences, The National University of Science and Technology, Bulawayo 029, Zimbabwe; 4 Faculty of Medicine and Health Sciences, Midlands State University, P Bag 9005, Gweru 054, Zimbabwe; 5 The Union Zimbabwe Trust, Harare 024, Zimbabwe; 6 Ministry of Health and Child Care, Harare 024, Zimbabwe; 7 Family Medicine, Global and Public Health Unit, Department of Primary Health Care Sciences, University of Zimbabwe, Harare 024, Zimbabwe; 8 Jointed Hands Welfare Organization, Gweru 054, Zimbabwe; 9 Hospice and Palliative Care Association of Zimbabwe, Harare 054, Zimbabwe; 10 Department of Health Sciences, Faculty of Sciences, Zimbabwe Open University, Harare 024, Zimbabwe

**Keywords:** artisanal miners, silicosis, silicotuberculosis, HIV, silica exposures

## Abstract

Artisanal and small-scale mining is characterized by an excessive exposure to silica-containing dust, overcrowding, poor living conditions and limited access to primary health services. This poses a risk to tuberculosis, HIV infection and silicosis. The main purpose of the study is to evaluate the burden of tuberculosis, HIV and silicosis among artisanal and small-scale miners. We conducted a cross sectional study on 3821 artisanal and small-scale miners. We found a high burden of silicosis (19%), tuberculosis (6.8%) and HIV (18%) in a relatively young population, with the mean age of 35.5 years. Men were 1.8 times more likely to be diagnosed with silicosis compared to women, adjusted prevalence ratio [aPR = 1.75 (95% CI: 1.02–2.74)]. Artisanal and small-scale miners who were living with HIV were 1.25 times more likely to be diagnosed with silicosis compared to those who were negative, [aPR = 1.25 (1.00–1.57)]. The risk of silicosis increased with both duration as a miner and severity of exposure to silica dust. The risk of tuberculosis increased with the duration as a miner. Zimbabwe is currently experiencing a high burden of TB, silicosis and HIV among artisanal and small-scale miners. Multi-sectoral and innovative interventions are required to stem this triple epidemic in Zimbabwe.

## 1. Introduction

Artisanal and small-scale mining (ASSM) occurs in over approximately 80 countries world-wide [1]. In 2017, an estimated 40.5 million people were directly engaged in ASSM and, of these, 10 million were living in 40 countries in sub-Saharan Africa [1,2,3]. Zimbabwe has over 500,000 people engaged in ASSM with over two million people dependent on it [4].

ASSM is associated with serious occupational health concerns that require adequate attention. These arise owing to ASSM’s labor intensiveness and un-mechanized mining methods, simple tools and equipment that result in low mineral yields associated with high exposures to silica-containing dust [5]. As a result, the majority of artisanal and small-scale miners (ASMs) are very poor, and work in risky working environments with multiple occupational hazards such as silica dust, noise, cyanide, and mercury among many others [6]. There is currently no compensation scheme for occupational diseases and injuries that covers ASMs in Zimbabwe. Frequent conditions observed in ASSM activity zones include but are not to limited to malaria, human immunodeficiency virus/acquired immunity deficiency syndrome (HIV/AIDS), tuberculosis (TB), malnutrition, mercury neurotoxicity and silicosis [7].

There is poor access to primary and occupational health (OH) services in remote ASSM communities. ASMs lack awareness on occupational hazards, and implementation of the hierarchy of controls to manage these hazards is often absent. This is compounded by their informal, illegal status, high mobility and unregulated nature of their activities, with inadequate or lacking safety measures [8,9], creating barriers to accessing health care including government health and surveillance programs [10]. As a result, critical environmental concerns such as mercury pollution, poor sanitation, lack of clean water and exposure to respirable crystalline silica remain unchecked. Policy reforms and initiatives to implement the hierarchy of controls by the ASM associations in collaboration with governments are urgently needed to address the poor health and safety standards, living standards and mining methods in ASSM.

Several studies have demonstrated the multiplicative risk of TB in HIV-positive miners diagnosed with silicosis [11,12,13,14,15]. Silica-containing dust with or without silicosis increases the risk of TB by three- to four-fold. Subjects with both silicosis and HIV have more than a fifteen-fold risk of developing TB [16]. Silica-containing dust is associated with multiple health conditions that include renal failure, scleroderma, systemic lupus erythematosus, sarcoidosis, chronic obstructive pulmonary disease, silicosis, lung cancer, rheumatoid arthritis and Erasmus Syndrome [17]. Notably, there is a rising epidemic of silicosis in southern Africa, a condition regrettably permanent and incurable [18]. Furthermore, the emergence of new evidence on ultra-fine and nano-sized silica particles with possible extra-pulmonary effects on other organs poses a significant risk to the health of miners [19]. The current diagnostic and surveillance methods can only detect silicosis in the advanced stages, presenting difficulties in the management of silicosis. The increasing burden of silicosis that is poorly documented among ASMs poses a significant risk to TB infection. There are currently no conclusive biomarkers that can detect the disease in the preclinical stages [20]. Primary prevention and implementation of the hierarchy of controls are grossly suboptimal among ASMs and the burden of silicosis and TB is substantial.

There is a dearth of published literature on the burden of occupational lung diseases (OLD) and TB among ASMs. The International Labour Organization (ILO) and World Health Organization (WHO) established the ILO/WHO Global Programme for the Elimination of Silicosis (GPES) following the recommendations of the 1995 joint ILO/WHO Committee on Occupational Health [21]. Global efforts to address occupational related morbidity and mortality have been prioritized in the current United Nations 2030 Agenda for Sustainable Development with Sustainable Development Goals (SDG 3.9) targeting to substantially reduce the number of deaths and illnesses from hazardous chemicals and air, water and soil pollution and contamination; additionally, SDG 8.8 is targeting to protect labor rights for all workers [22].

From 2017 to date, Zimbabwe has implemented screenings for OLD in ASMs under the Global Fund’s “TB in the mining sector” and the United States Agency for International Development (USAID)’s Kunda Nqob’iTB (KNTB) projects [23,24]. The USAID project adopted an OH approach that screens for TB, HIV, silicosis and silico-tuberculosis through dedicated OH clinics and medical out-reach facilities covering TB hotspots characterized by high ASSM activities [25]. Health worker capacity building on TB and OLD diagnosis was also implemented as part of the OH package. With the hypothesis that ASMs who use rudimentary mining methods are heavily exposed to silica dust resulting in higher TB and silicosis levels. The project published work on “TB and silicosis Burden in Artisanal and Small-Scale Gold Miners in a large OH Outreach Programme in Zimbabwe, 2021” in which the prevalence of silicosis and TB were 11.2% and 4%, respectively, among ASMs [9]. However, this was based on a small sample size of 514 ASM medical records and was from one OH clinic in Gweru. This could have undermined the true burden of silicosis, TB and HIV. Therefore, this study sought to determine the prevalence of silicosis, HIV, TB and silico-TB from a larger sample size drawn from two OH clinics and medical outreach facilities in the Matabeleland south and Midlands provinces from 01 October 2020 to 31 May 2022.

The specific objectives of the study are to: (i) determine the yields and prevalence of silicosis, TB and HIV among ASMs from occupational health clinics and occupational health outreach facilities and (ii) evaluate factors associated with a diagnosis of silicosis, TB and HIV among ASMs.

## 2. Materials and Methods

### 2.1. Study Design

A cross-sectional retrospective review of the occupational health records of ASMs who were screened for silicosis, TB and HIV at two occupational health clinics and outreach mining sites in the Midlands and Matabeleland South provinces was conducted.

### 2.2. General Setting

**Figure 1:**
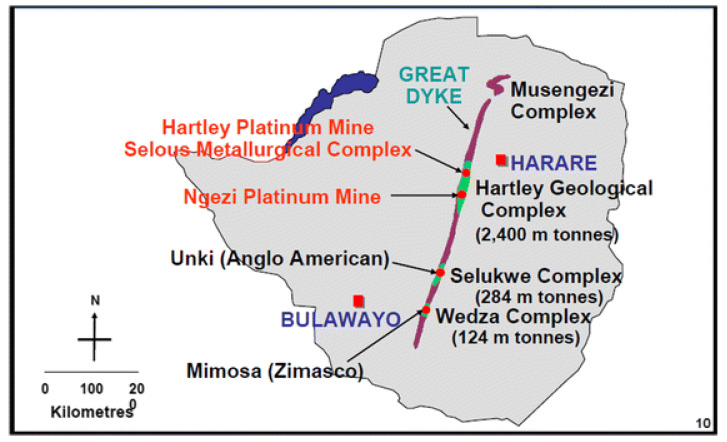
The Great Dyke.

Zimbabwe has a population of 15.2 million, spread across 10 provinces and 91 administrative districts [26]. Most mining activities occur along the Great Dyke which is approximately 550 km long and is 3 km–12 km wide as shown in Figure 1 [27]. The Great Dyke is rich in gold, diamonds, platinum, chrome, asbestos and tin [28]. Zimbabwe’s TB incidence rate declined from 242 per 100,000 in 2015 to a rate of 199 per 100,000 for 2019 [29]. Zimbabwe has an HIV prevalence of 12.9% [30].

### 2.3. Specific Setting and Screening Procedure KNTB

The study was performed in seven KNTB supported districts from two provinces, Midlands and Matabeleland South. The districts included Zvishavane, Insiza, Gwanda, Gweru, Chirumhanzu, Kwekwe and Shurugwi of which the first four districts participated in the 2021 study [9].

#### 2.3.1. Screening and Diagnosis Method

The screening methodology used in the previous study was adopted where all ASMs were assessed for TB using a symptoms screen tool and chest radiographs. The diagnosis of TB was based on a positive Xpert mycobacterium tuberculosis rifampicin resistance (xMTB/RIF) Ultra assay (Cepheid, SunnyVale, CA, USA) and/or clinical findings as per national TB Guidelines [31]. Screening for silicosis was performed using chest radiographs (Apelem digital X-ray machine 2010 u, Apelem digital X-ray 2012, Apelem, Nimes, France [32], Genin digital X-ray 2020, Toronto, ON, Canada) at the two occupational health clinics and the mobile digital X-ray machine. Interpretations of the chest radiographs were performed by medical officers trained in the diagnosis of OLD and TB and were further checked by a specialist occupational physician experienced in the International Labour Organization (ILO)’s international classification of chest radiographs for the prevention of the pneumoconioses. The diagnostic criteria for silicosis were a bilateral multinodular pattern with or without progressive massive fibrosis on chest radiographs, a positive occupational history of exposure to silica-containing dust and having or not having symptoms of a subtle and progressive shortness of breath or cough, in the absence of any other identifiable disease/s [33]. A diagnosis of silicosis was based on a threshold profusion of ≥1/0 on the ILO classification of chest radiographs [34]. The diagnosis of silico-TB was based on the presence of both silicosis and TB. Since the study was conducted during the COVID-19 pandemic, spirometry testing was not conducted as it is an aerosol-generating procedure. Data on ASMs were captured in presumptive TB registers and occupational health records. During mobile outreaches, presumptive TB registers that were used were obtained from participating clinics within the catchment area where outreaches were conducted. ASMs who were diagnosed with TB and/or silicosis were entered in TB and TB-preventive therapy registers, respectively, and were linked to care through the local health facilities.

Voluntary counselling and testing for HIV were offered to all ASMs during TB and silicosis screening. The HIV status was based on the Oraquick self-test (OraSure technologies Inc., Bethlehem, Philadelphia, PA, USA), Determine and Chembio HIV test kits, documented previous test results, and self-reported HIV statuses. Those who had a reactive Oraquick test were referred to the opportunistic infection clinic for confirmatory HIV testing according to WHO HIV testing guidelines [35]. Some ASMs came with valid HIV results and others self-reported their HIV status.

#### 2.3.2. Occupational Health Clinic Procedures

Under the USAID KNTB-funded project, two occupational health clinics were set up within the premises of Gwanda and Gweru provincial hospitals, which are public health institutions. The occupational health clinics were set up to serve ASMs and provide free TB and OLD screening services to ASMs who are either well or unwell and voluntarily present to the unit. Staff working at these OH clinics were trained in the diagnosis and management of TB and OLDs and are supported by a specialist occupational physician. Demand creation for the occupational health clinics is performed by community mobilisers and focal ASMs who provide awareness on services offered at the facility. The OH clinics attend to any ASMs seeking services, including those referred from other facilities or from within the local facility where they are located.

##### Workflow Process at the OH Clinic

At the OH clinics, ASMs are offered health and safety education on TB, occupational hazards and diseases associated with silica dust exposure. Routine observations, a full health assessment including a physical examination, chest radiographs and sputum examination for TB are performed at the institution. All ASMs undergo symptom screening and chest radiography, and only those with TB symptoms and/ or abnormal chest radiographs submit a sputum for testing using the xMTB/RIF Ultra assay. Voluntary HIV testing and counselling is offered to all ASMs. ASMs diagnosed with TB, or any other medical conditions are referred to the relevant service points at the hospital. Those diagnosed with silicosis are evaluated for TB Preventive Therapy (TPT) eligibility. The regimens used for TPT are 6 months of daily Isoniazid (6H) or 3 months of weekly doses of isoniazid and rifapentin (3HP) [31]. Data collection and recording is performed through the health institution’s data tools.

#### 2.3.3. Outreach Procedure

Under the USAID’s KNTB project, an occupational health outreach facility provides TB and OLD services to ASMs at the mining sites. ASM sites include both underground and surface mining. The mobile outreach unit consists of a truck equipped with a digital X-ray machine. A team of doctors and other health care workers from the local clinic or hospital provides the same services described under the OH clinics. Sputum samples for TB are collected on site and transported the same day to the nearest testing laboratory for xMTB-RIF Ultra assay as per Ministry of Health and Childcare (MOHCC) protocols [36]. All cases of TB, HIV, silicosis and other diseases are linked to care at the local hospitals and clinics for treatment and follow-up. All clinical data are collected and recorded in the MOHCC registers of the participating clinics or hospitals. The mobile outreaches are conducted over a four-day period in each project district once a year.

Sites for screening at the ASSM areas are selected on the basis of high ASM activity in the area. Prior to screening, a two-day mobilization exercise is conducted at least a week in advance to sensitize the miners, establish rapport and identify a camping site that is at the mining sites or very close to the mining sites. The site selection and awareness activities are conducted by the medical team, the local ASM leadership and the TB focal person from the nearest health facility. This team also liaises with the local leadership to support the outreach activity. The mobile unit camps at or close to the ASM sites whereupon attendance is voluntary. The workflow process during outreaches is the same as at the OH clinics.

### 2.4. Study Population

The study population consisted of ASMs working in the KNTB supported districts except Mwenezi. The study population comprised ASMs (men and women) who were involved in the extraction and/or processing of gold or chrome either underground or surface mining presenting at the OH clinics or during outreach screening activities between 1 October 2020 and 31 May 2022. The ASMs included those working in underground and/or surface mines as individuals, in family groups, in partnerships or as members of cooperatives.

### 2.5. Inclusion Criteria

All complete occupational health records of ASMs who were attended to at the OH clinics and medical outreach facility were included in the study. The records containing complete demographic details, routine observations and tests such as chest X-ray, HIV and xMTB-RIF Ultra assay results were included in the study.

### 2.6. Exclusion Criteria

Occupational health records with missing chest radiograph results and demographic data were excluded from the study. The records of ASMs seen from outside the stipulated project districts were excluded from the study.

### 2.7. Sampling Procedure

All the available 3950 medical records of ASMs that were available during the study period were included in the study. This sample size was higher than the 514 records reviewed in the previous study [9].

### 2.8. Data Variables, Sources of Data and Data Collection

Individual level data were extracted from occupational health records of ASMs screened at the two medical facilities and at outreaches. The data variables included age, gender, HIV status, history of TB, pneumonia, asthma and chronic cough, exposure to silica dust, duration of mining, substance use and alcohol use. The assessment of exposure to silica dust was subjective and based on whether the ASMs perceived themselves as being exposed to dust or not during mining. Data were collected using a pre-coded data proforma.

### 2.9. Ethical Approval

The permission to access records was obtained from the Secretary for Health and Child Care, Ministry of Health and Child Care, Zimbabwe. Permission to conduct the study was also obtained from the Zimbabwe Artisanal and Smale-Scale Miners’ Association. The ethics approval was obtained from the Medical Research Council of Zimbabwe (MRCZ) (Approval number: MRCZ/E/307).

### 2.10. Data Analysis

Data were individually entered in Epidata Entry software (EpiData Association, Odense, Denmark) and were exported to Stata v 13.0 (Stata Corporation, College Station, TX, USA) for cleaning and analysis [37]. Categorical variables were summarized using proportions, while continuous variables were summarized using means and standard deviations as appropriate. The differences between means were assessed using the independent *t*-test. The key outcome variables were silicosis, TB, HIV and silico-TB diagnosis. The effect of predictor variables on each of the dependent (outcome) variables was investigated using binomial regression. Poisson regression was performed to investigate the association between predictor variables and the key outcome variables after adjusting for the following confounders: age, sex, duration as a miner, level of exposure to silica, comorbidities, HIV status and previous history of TB. The associations were expressed as prevalence ratios (PRs) and adjusted PRs. The level of significance was set at 5% (*p* < 0.05).

## 3. Results

### 3.1. Sociodemographic and Clinical Characteristics

From a total of 3950 medical records of ASMs reached, 3821 records that met the eligibility criteria were selected. The socio-demographic and clinical characteristics of the ASMs are shown in Table 1. Overall, there were 3243 (85%) men. The modal age category was 25–34 years, which accounted for 1208 (32%) of the study participants. The mean age and standard deviation (SD) among ASMs who visited occupational health facilities was 36.9 (12.2). This was higher than the mean age of 35.0 (12.0) among ASMs who attended outreach facilities, *p* = 0.001. At least three-quarters had attained secondary education. Of the 2568 ASMs who had a known HIV status, 460 (18%) were HIV-positive. At least a quarter had ≥10 years’ duration as miners. ASMs who visited OH clinics were more likely to report a history of TB (20% versus 6%) and comorbidities, e.g., pneumonia (17% versus 4%) compared to those who visited outreach facilities, respectively as shown in Table 2.

### 3.2. Silicosis among ASMs

The factors associated with silicosis diagnosis are shown in Table 3. Overall, 666 ASMs [19% (95% CI: 17.5–20.1)] were diagnosed with silicosis. Men were 1.8 times more likely to be diagnosed with silicosis compared to women, adjusted prevalence ratio [aPR = 1.75 (95% CI: 1.02–2.74)]. ASMs who were living with HIV were 1.25 times more likely to be diagnosed with silicosis compared to those who were negative, [aPR = 1.25 (1.00–1.57)]. The risk of silicosis increased with both duration as a miner and severity of exposure to silica dust.

### 3.3. TB among ASMs

Overall, the number of ASMs diagnosed with TB was 240 [6.7% (95% CI: 6.0–7.6)] as shown in Table 4. This translates to a prevalence rate of 6766 per 100,000 populations. The risk of TB increased with severity of exposure to dust, 2.66 times higher in ASMs who reported severe exposures versus those who reported mild exposures and was 8.72 times higher among ASMs who attended the occupational health clinics, [aPR = 8.72 (95% CI: 6.10–12.5)]. For TB cases, the number needed to screen (NNS) at the occupational health clinics was 5 (*n* = 956/190) compared to 52 (*n* = 2591/50) at medical outreach facilities.

### 3.4. Silico-TB among ASMs

Overall, 89 [2.5% (95%CI: 2.0–3.1)] ASMs were diagnosed with silico-TB. The risk factors for silico-TB included a history of TB, pneumonia, alcohol intake and age category of 35–44 years as shown in Table 5 below. ASMs who had been mining for ≥10 years were 2.60 times more likely to have silico-TB compared to ASMs who had a mining experience of <5 years, [aPR = 2.56 (95% CI:1.12–5.86)].

## 4. Discussion

In this large-scale study, we aimed to determine the prevalence of silicosis, TB and HIV among ASMs. We found a high prevalence of silicosis, HIV and TB. The risk of silicosis and TB increased with the duration and intensity of exposure to silica dust; duration as a miner and exposure was high in the age category of 35–44 years.

The prevalence of silicosis was comparable to studies conducted in South Africa [13,38] but was higher than the prevalence of 11.7% in an earlier smaller study in Zimbabwe [9]. In this study, ASMs were older and had a higher duration as miners compared to ASMs who were enrolled in the previous study [9]. That the risk of silicosis increases with age and intensity of exposure is a finding that has been confirmed by this study. This perhaps explains why our reported prevalence was lower than the prevalence of 26.6% in Botswana [39], 37% in Brazil [14], 42% in Lesotho [40] and 34% in Transkei [41], South Africa. The Lesotho study enrolled older ex-underground miners. The risk of silicosis is expected to be high among ex-miners due to longer periods of exposure. Studies have reported an increased risk of developing silicosis even after exposure to silica has ceased [15,42,43].

Our study has confirmed the association between silicosis and HIV infection among ASMs, similar to our previous study [9]. This finding is crucial given that Zimbabwe is an HIV-burdened setting. ASMs are disproportionally affected since the prevalence of 18% is higher than the national HIV prevalence of 12.9% [30]. Of note, just over a third (1253 ASMs) either opted out of HIV testing or did not consent to provider-initiated HIV testing. One hundred and seventy-seven (27%) of the silicosis cases had an unknown HIV status, which indicates a possible underestimation of the actual HIV burden in this population. It is plausible that ASMs who refused HIV testing may have engaged in risky sexual behaviors for HIV.

A high prevalence of TB among ASMs in Zimbabwe was described by Moyo et. al., 2021 [9]. This study, together with studies conducted in other countries, has shown a high prevalence of TB among ASMs in Zimbabwe, Malawi (14%), Ghana (4.2%) and Tanzania (8.3%) [44,45,46]. Silica dust, silicosis and HIV infection are risk factors for TB infection [13,14,15,42]. In this study we observed a triple burden of silicosis, TB and HIV. This may fuel the TB epidemic in Zimbabwe in several ways. Firstly, the large population of ASMs is exposed to high silica dust levels with a near absent application of the hierarchy of controls in reducing dust exposures. Secondly, the high mobility of ASMs and their poor health seeking behaviors are likely to promote the spread of TB within mining populations and the general public. Thirdly, a lack of adequate human resources with skills and experience in the diagnosis of OLD such as silicosis and initiation of TB preventive therapy is a challenge. Fourthly, the diagnosis of TB in the presence of silicosis is often difficult due to the similarity and overlap of symptoms. The fibrosis of silicosis prevents discharge of mycobacteria tuberculosis into sputum specimens making its detection difficult [47].

Our study is strengthened by the enrolment of a larger sample size, including an additional four gold and/or chrome mining districts, compared to our previous study that had a limited sample size from only four gold mining districts. Our study further confirmed our previous findings of the association between HIV and silicosis. Furthermore, our study has confirmed the high prevalence of silicosis (19%), TB (6.8%) and HIV (18%), which was reported in our previous smaller study: silicosis (11.2%), TB (4%) and HIV (23.5%) [9]. Following the first study, there has been training among medical officers and nurses and a better awareness of TB and silicosis screening. This may have increased demand for screening services by ASMs. The demand was matched by enhanced capacity among healthcare providers in screening for silicosis, TB and HIV. We limited sampling bias by including all records for ASMs who attended the OH clinics and outreach facilities.

Our study, however, had some limitations. Firstly, we cannot rule out bias related to the Healthy Worker Effect. During the mobile outreach screenings, we enrolled ASMs who were actively mining. As such those who were sick were less likely to have been available. Secondly, ASMs who died or left the ASM pool due to ill health were not enrolled. Sicker ASMs or those who die are likely to have a higher proportion of silicosis and TB than those who were actively mining. Thus, the prevalence reported here could have been underestimated. Lastly, our study population was confined to 2 out of 10 the provinces in Zimbabwe. This may affect the generalizability of our findings to the whole population of ASMs in Zimbabwe. Furthermore, in some cases, the HIV status was self-reported, and this could have affected the overall HIV prevalence in this study.

Despite these limitations, this first large-scale study combining outreach and occupational health clinic screening in a key population group of ASMs for silicosis and TB has some important implications for policy considerations. While some studies focused on identifying hazards among ASMs, this study focused on identifying diseases [8]. The triple burden of silicosis, TB and HIV among economically productive age groups of ASMs heightens attention to multi-sectoral collaborations among the Zimbabwe Miners Federation, Ministry of Mines, Ministry of Public Service, Labor and Social Welfare through the National Social Security Authority (NSSA) and AIDS and TB programs to tackle this growing epidemic in Zimbabwe.

While silicosis is preventable, there is no effective treatment. People who are no longer exposed to silica may still be at risk of developing silica-related diseases, the risk increases with both the duration and intensity of exposure to silica. In formal mining, once silicosis is diagnosed, workers are given tasks where they are less exposed to silica dust, and they are compensated. This is a challenge where informal mining is done. It is also difficult to enforce the hierarchy of control measures to reduce dust levels.

Regular screening of ASMs for silicosis and the timely initiation of TB preventive therapy (TPT) for those diagnosed with silicosis is required. TPT reduces the prevalence of and timely initiation in ASMs with silicosis can reduce the development and spread of TB [48,49]. As a result, there is no legislation that supports such screening. There is the need to register and regularize the operations of ASMs so that some legislation such as the Pneumoconiosis Act and Statutory 68 of 2000 can be applied to ensure the regular screening of ASMs in Zimbabwe [50,51]. There is also the need for dedicated occupational health clinics in districts with high ASM activity. This should be complemented by interventions that are tailored to the needs of highly mobile ASMs.

## 5. Conclusions

We have described a high burden of TB, silicosis and HIV among ASMs. These conditions require regular contact with the healthcare system by ASMs―a key population group that has poor access to healthcare services and poor health seeking behaviors. Multi-sectoral and innovative interventions are required to stem this triple epidemic in Zimbabwe. Strategies aimed at reducing the exposure to silica-containing dust among ASMs remain paramount in lowering or eliminating the risk of TB and silicosis.

## Figures and Tables

**Table 1:** Socio-demographic characteristics among artisanal and small-scale miners in Zimbabwe.

Characteristic		Type of Facility				Total	
		Outreach		OH Clinic			
		2744	(%) ^†^	1077	(%) ^†^	3821	
Sex	Male	2381	(87)	862	(80)	3243	(85)
	Female	347	(13)	211	(20)	558	(15)
	Not recorded	16	(<1)	4	(<1)	20	(<1)
Age (years)	10–19	117	(4)	40	(4)	157	(4)
	20–24	470	(17)	131	(12)	601	(16)
	25–34	881	(32)	327	(30)	1208	(32)
	45–54	691	(25)	326	(30)	1017	(27)
	55+	575	(21)	246	(23)	821	(21)
	Not recorded	10	(<1)	7	(1)	17	(<1)
Mean (SD)		35.0 (12.0)		36.9 (12.2)		35.5 (12.1)	
Education	None	27	(1)	29	(3)	56	(1)
	Primary	490	(18)	205	(19)	695	(18)
	Secondary	2082	(76)	797	(74)	2879	(75)
	Tertiary	109	(4)	30	(3)	139	(4)
	Not recorded	36	(1)	16	(1)	52	(2)
HIV status	Positive	242	(9)	218	(20)	460	(12)
	Negative	1503	(55)	605	(56)	2108	(55)
	Unknown	999	(36)	254	(24)	1253	(33)
District	Shurugwi	854	(31)	381	(35)	1235	(32)
	Gwanda	402	(15)	264	(25)	666	(17)
	Gweru	266	(10)	339	(31)	605	(16)
	Kwekwe	459	(17)	57	(5)	516	(14)
	Insiza	396	(14)	5	(0)	401	(10)
	Chirumhanzu	351	(13)	8	(1)	359	(9)
	Zvishavane	16	(1)	23	(2)	39	(1)
Duration as a miner	<5	1389	(51)	440	(41)	1829	(48)
	5.0–9.9	616	(22)	240	(22)	856	(23)
	10+	677	(25)	368	(34)	1045	(27)
	Not recorded	62	(2)	29	(3)	91	(2)
	Median (IQR)	4.75 (2.00–9.75)		6.00 (2.75–11.75)		5.0 (2.8–10.8)	

^†^ = Column percentages; OHC = Occupational health clinic; SD = standard deviation; IQR = Interquartile range.

**Table 2:** Comorbidities among artisanal and small-scale miners in Zimbabwe.

Characteristic		Type of Facility			Total		
		Outreach		OHC			
		2744	(%) ^†^	1077	(%) ^†^		
Substance use	Yes	467	(17)	206	(19)	673	(18)
	No	2242	(82)	856	(80)	3098	(81)
	Unknown	15	(1)	15	(1)	50	(1)
Alcohol use	Yes	1328	(51)	463	(43)	1791	(47)
	No	1392	(48)	611	(57)	2003	(52)
	Not recorded	24	(1)	3	(<1)	27	(1)
Exposure to silica	Severe	1500	(55)	810	(75)	2310	(12)
	Moderate	693	(25)	155	(14)	848	(22)
	Mild	361	(13)	84	(8)	445	(60)
	Not recorded	190	(7)	28	(3)	218	(6)
Asthma	Yes	42	(2)	25	(2)	67	(2)
	No	2541	(92)	1052	(98)	3593	(94)
	Not recorded	161	(6)	-	-	161	(4)
Chest injury	Yes	31	(1)	12	(1)	43	(1)
	No	2154	(79)	1064	(99)	3218	(84)
	Not recorded	559	(20)	1	(<1)	560	(15)
Pneumonia	Yes	116	(4)	187	(17)	303	(8)
	No	2163	(79)	889	(83)	3052	(80)
	Unknown	465	(17)	1	(<1)	466	(12)
History of TB	Yes	167	(6)	217	(20)	384	(10)
	No	2552	(93)	860	(80)	3412	(89)
	Not recorded	25	(1)	-	-	25	(1)

^†^ = Column percentages; OHC = Occupational health clinic.

**Table 3:** Factors associated with diagnosis of silicosis.

Characteristic		Total	Diagnosed with Silicosis		PR (95% CI)	aPR (95% CI)
Overall		3547	666	(19) ^‡^		
Sex (*n* = 3529)	Male	3029	622	(21)	2.50 (1.85–3.39) *	1.78 (1.02–2.74) *
	Female	500	41	(8)	Reference	Reference
Age (years) (*n* = 3533)	10–19	142	4	(3)	0.40 (0.14–1.10)	0.42 (0.13–1.40)
	20–24	566	40	(7)	Reference	Reference
	25–34	1128	176	(16)	2.21 (1.59–3.07) *	1.29 (0.84–2.00)
	35–44	948	233	(25)	3.48 (2.53–4.78) *	1.55 (1.00–2.43)
	45+	749	211	(28)	3.99 (2.89–5.49) *	1.57 (0.99–2.48)
Education (*n* = 3501)	None	50	15	(30)	4.65 (2.10–10.27) *	3.64 (1.25–10.6) *
	Primary	642	163	(25)	3.94 (1.99–7.79) *	3.18 (1.29–7.84) *
	Secondary	2685	467	(17)	2.70 (1.37–5.30) *	2.41 (0.99–5.85)
	Tertiary	124	8	(6)	Reference	Reference
HIV status (*n* = 2442)	Positive	437	141	(32)	1.86 (1.57–2.19) *	1.25 (1.00–1.57) *
	Negative	2005	348	(17)	Reference	Reference
Duration as miner (*n* = 3547)	<5	1785	168	(9.4)	Reference	Reference
	5.0–9.9	797	162	(20.3)	2.16 (1.77–2.64) *	2.11 (1.58–2.83) *
	10–40	965	336	(34.8)	3.70 (3.13–4.38) *	2.59 (1.96–3.42) *
PPE (*n* = 3520)	No	2517	503	(20.0)	1.27 (1.08–1.49) *	1.21 (0.98–1.52)
	Yes	1003	158	(15.8)	Reference	Reference
Asthma (*n* = 3394)	Yes	64	7	(10.9)	0.57 (0.28–1.15)	0.61 (0.27–1.38)
	No	3330	639	(19.2)	Reference	Reference
Alcohol intake (*n* = 3525)	Yes	1654	345	(20.9)	1.25 (1.09–1.43) *	1.07 (0.88–1.30)
	No	1871	313	(16.7)	Reference	Reference
History of TB (*n* = 3526)	Yes	347	157	(45.2)	2.87 (2.49–3.30) *	1.68 (1.33–2.13) *
	No	3179	502	(15.8)	Reference	Reference
Pneumonia (*n* = 3059)	Yes	269	78	(29.0)	1.51 (1.23–1.85) *	1.33 (1.04–1.71) *
	No	2830	543	(19.2)	Reference	Reference
Exposure to silica (*n* = 3350)	Severe	2139	480	(22.4)	2.38 (1.74–3.24) *	1.89 (1.33–2.91) *
	Moderate	798	118	(14.8)	1.57 (1.11–2.20) *	1.45 (0.99–2.14)
	Mild	413	39	(9.4)	Reference	Reference
						
Chest surgery (*n* = 3016)	Yes	41	15	(36.6)	1.8 (1.2–2.8) *	1.28 (0.60–2.71)
	No	2975	592	(19.9)	Reference	Reference
Type of facility (*n* = 3547)	Occupational	956	239	(25)	1.52 (1.32–1.74) *	1.04 (0.85–1.29)
	Outreach	2591	427	(17)	Reference	Reference

^‡^ = row percentages; PR = prevalence ratio; aPR = adjusted prevalence ratio; CI = Confidence interval. * = Statistically significant

**Table 4:** Factors associated with TB diagnosis among artisanal and small-scale miners in Zimbabwe.

Characteristic		Total	Diagnosed with TB		PR (95% CI)	aPR (95% CI)
Overall		3547	240	(6.8)		
Sex (*n* = 3529)	Male	3029	226	(7.5)	2.9 (1.7–5.0) *	2.84 (1.57–5.48) *
	Female	500	13	(2.6)	Reference	Reference
Age (years) (*n* = 3527)	10–19	136	2	(1.5)	0.7 (0.2–3.1)	1.27 (0.26–6.18)
	20–24	566	12	(2.1)	Reference	Reference
	25–34	1128	73	(6.5)	3.2 (1.62–28.39) *	2.83 (1.26–6.33) *
	35–44	948	102	(10.8)	5.4 (2.90–49.22) *	3.53 (1.57–7.93) *
	45+	749	50	(6.7)	3.3 (1.9–6.0) *	2.38 (1.02–5.57) *
Education (*n* = 3501)	None	50	9	(18.0)	2.9 (1.6–5.3) *	1.51 (0.74–3.11)
	Primary	642	53	(8.3)	1.3 (1.0–1.8)	1.08 (0.77–1.52)
	Secondary	2685	169	(6.3)	Reference	Reference
	Tertiary	124	2	(1.6)	0.3 (0.1–1.0)	0.23 (0.03–1.68)
HIV status (*n* = 2442)	Positive	437	72	(16.5)	2.8 (2.2–3.8) *	1.51 (1.10–2.10) *
	Negative	2005	116	(5.8)	Reference	Reference
Exposure to silica (*n* = 3350)	Severe	2139	194	(9.1)	5.4 (2.5–11.3) *	2.66 (1.08–6.55) *
	Moderate	798	32	(4.0)	2.4 (1.1–5.3) *	1.97 (0.75–5.16)
	Mild	413	7	(1.7)	Reference	Reference
History of TB (*n* = 3526)	Yes	347	77	(22.2)	4.4 (3.4–5.6) *	1.75 (1.30–2.37) *
	No	3179	162	(5.1)	Reference	Reference
Pneumonia (*n* = 3059)	Yes	269	60	(22.3)	3.82 (2.92–5.00) *	2.04 (1.49–2.79) *
	No	2830	165	(5.8)	Reference	Reference
Asthma (*n* = 3394)	Yes	64	6	(9.4)	1.4 (0.60–2.9)	1.39 (0.62–3.22)
	No	3330	229	(6.9)	Reference	Reference
Alcohol intake (*n* = 3525)	Yes	1654	132	(8.0)	1.4 (1.1–1.8) *	1.34 (1.02–1.77) *
	No	1871	105	(5.6	Reference	Reference
Duration as miner (*n* = 3465)	<5 years	1703	55	(3.2)	Reference	Reference
	5.0–9.9 years	797	61	(7.7)	2.4 (1.7–3.4) *	1.56 (1.05–2.31) *
	10–40 years	965	116	(12.0)	3.7 (2.7–5.1) *	1.77 (1.22–2.57) *
Type of facility (*n* = 3547)	Occupational	956	190	(19.9)	10.3 (7.6–14.0) *	8.72 (6.10–12.5) *
	Outreach	2591	50	(1.9)	Reference	Reference

PR = prevalence ratio; aPR = adjusted prevalence ratio; CI = Confidence interval; * = Statistically significant.

**Table 5:** Factors associated with silico-TB diagnosis among artisanal and small-scale miners in Zimbabwe.

Characteristic		Total	Diagnosed with Silico-TB		PR (95% CI)	aPR (95% CI)
Overall		3547	89	(2.5) ^‡^		
Sex (*n* = 3529)	Male	3029	84	(2.8)	2.77 (1.13–6.80) *	2.74 (0.64–11.8)
	Female	500	5	(1.0)	Reference	Reference
Age (years) (*n* = 3533)	10–19	142	1	(0.7)	1.99 (0.18–21.83) *	2.57 (0.23–28.5)
	20–24	566	2	(0.4)	Reference	Reference
	25–34	1128	27	(2.4)	6.77 (1.62–28.39) *	4.34 (0.98–18.9)
	35–44	948	40	(4.2)	11.94 (2.90–49.22) *	5.46 (1.24–24.1) *
	45+	749	19	(2.5)	7.18 (1.68–30.70) *	3.79 (0.82–17.5)
HIV status (*n* = 2442)	Positive	437	20	(4.6)	2.41 (1.42–4.11) *	1.19 (0.72–2.34)
	Negative	2005	38	(1.9)	Reference	Reference
Exposure to silica (*n* = 3350)	Severe	2139	75	(3.5)	7.24 (1.78–29.37) *	
	Moderate	798	11	(1.4)	2.85 (0.63–12.78)	
	Mild	413	2	(0.5)	Reference	
History of TB (*n* = 3526)	Yes	347	30	(8.6)	4.66 (3.04–7.13) *	2.05 (1.14–3.67) *
	No	3179	59	(1.9)	Reference	Reference
Pneumonia (*n* = 3059)	Yes	269	21	(7.8)	3.40 (2.11–5.47) *	1.96 (1.17–3.29) *
	No	2830	65	(2.3)	Reference	Reference
Asthma (*n* = 3394)	Yes	64	3	(4.7)	1.84 (0.60–5.65)	3.00 (0.89–10.1)
	No	3330	85	(2.6)	Reference	Reference
Alcohol intake (*n* = 3525)	Yes	1654	60	(3.6)	2.61 (1.66–4.12) *	2.79 (1.51–5.14) *
	No	1871	26	(1.4)	Reference	Reference
Chest injuries (*n* = 3016)	Yes	41	4	(9.8)	3.5 (1.4–9.2) *	1.42 (0.47–4.24)
	No	2975	82	(2.8)	Reference	Reference
Duration as miner (*n* = 3547)	<5 years	1758	17	(1.9)	Reference	Reference
	5.0–9.9 years	797	23	(2.9)	1.93 (0.99–3.79)	1.87 (0.77–4.51)
	10–40 years	965	49	(5.1)	3.40 (1.86–6.23) *	2.56 (1.12–5.86) *
Type of facility (*n* = 3547)	Occupational	956	65	(6.8)	7.34 (4.62–11.65) *	7.67 (3.69–15.7) *
	Outreach	2591	24	(0.9)	Reference	Reference

^‡^ = row percentages; PR = prevalence ratio; aPR = adjusted prevalence ratio; CI = Confidence interval; * = Statistically significant.

## Data Availability

The data presented in this study are available on request from the corresponding author. The data are not publicly available due to authorizations that may be required by the Ministry of Health and Childcare of Zimbabwe.
